# Supplementary material for: Pysanky to Microfluidics: An Innovative Wax-Based Approach to Low Cost, Rapid Prototyping of Microfluidic Devices
Source: Micromachines (Basel). 2024 Feb 5;15(2):240. doi: 10.3390/mi15020240 (PMC10892862; doi:10.3390/mi15020240)
Supplement: Supplementary file 1 [file micromachines-15-00240-s001.zip › micromachines-2796043-supplementary.pdf]

Electronic Supplementary Information (ESI)

1. Traditional Ukrainian Easter Egg wax painting (Pysanky)

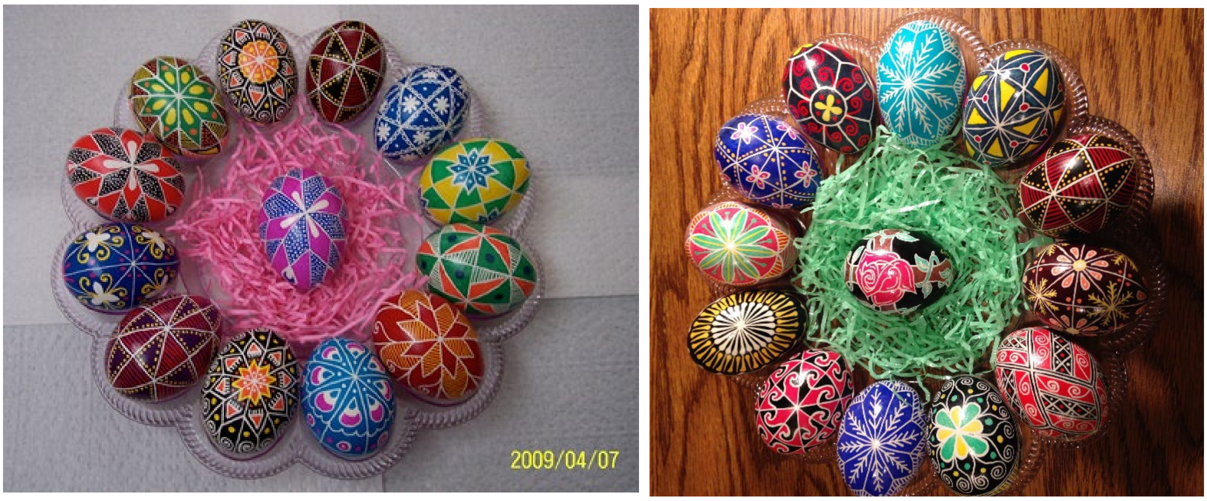

Figure S1 Traditional Ukrainian Easter eggs painted by local painter Lucy Piniarksi of West Seneca, NY. Each egg is hand painted using a wax kistka resulting in egg completion times ranging between 5 and 10 hours per egg. Traditional Ukrainian Easter egg painting is done by hand using a wax stylist called a kistka. There are a series of different size kistkas which allow the designer to choose between different volume output of the wax (i.e. for small intricate lines, or shading in an area with a lot of wax). Wax pellets are inserted in the backend of the kistka tip and then the tip is manually heated up by the designer, typically holding it over an open candle flame, until the wax reaches a low viscous state and begin to flow. The designer then draws on the surface of the egg covering it with wax. It should be noted that everything covered with wax remains that color, for instance, all the wax draw on the egg's surface will keep the covered part white. One can consider this wax as a masking material that preserves what is under it. The egg is then dipped in various color dyes and the process of wax design and dipping repeats. Once finished the wax is melted or dissolved off leaving behind the colourful patterned eggs.

2. System Setup

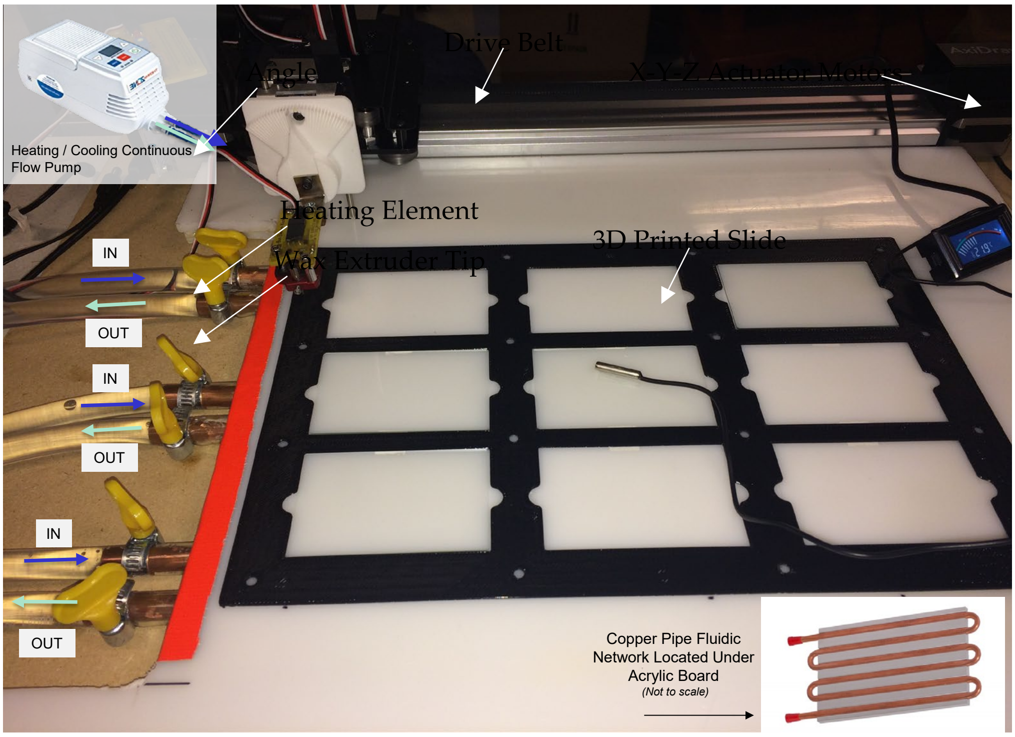

Figure S2 A system overview of the wax printing test setup. The original AxiDraw system, is versatile pen plotter capable of writing or drawing on flat surfaces. Similar to that of a 3D printer actuating extruder head, the AxiDraw has the ability to travel in the X-Y direction across an 11.81 by 8.58 inch surface. The motors have a resolution of 80 steps/mm and has the ability to retrace a previous pattern with a 0.1mm accuracy. The heating and cooling system consists of a series of copper pipes embed on the underside of an acrylic board. These pipes were sealed in with expanding insulating foam on the underside to prevent heat loss. The pipes were connected to a heating and cooling flow pump which provided continuous temperature controlled fluid flow. A 3D printed slide holder was created out of ABS plastic and can hold up to 9 different slides. The X-Y-Z actuator step motors controlled the drive belt which translated the wax extruder tip across the substrate. The wax extruder tip was connected to a heating element to maintain the wax temperature at a viscous state. A 3D printed compass was printed allowing for the contact angle between the wax extruder tip and the substrate to be controlled in 2° increments in either direction. It should be noted that both the external room temperature and humidity was recorded during each experiment, however due to a semi-control testing environment there was no significant variation.

3. Video clip 1_Serial Dilution Printing

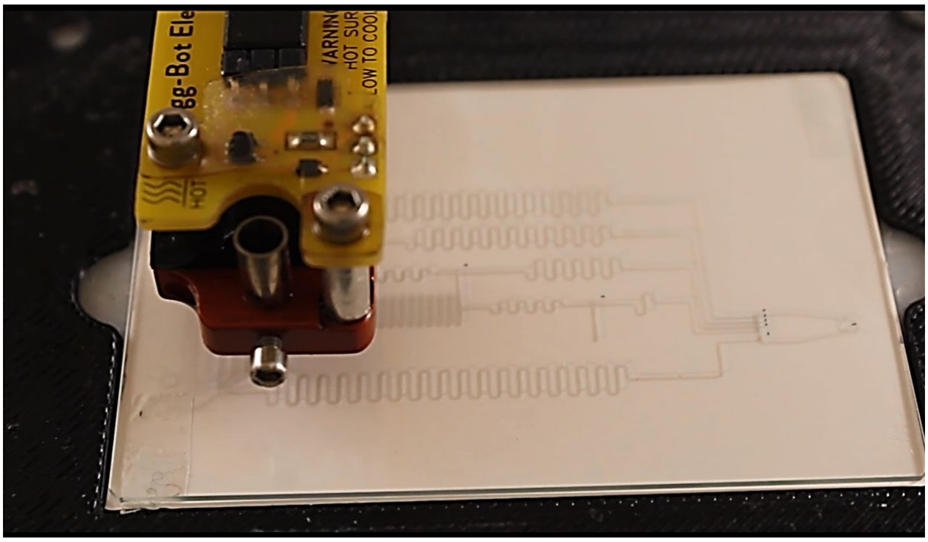

Figure S3 A screen capture from the video clip 1 (Video S1), during the wax printing process of the serial dilutor microfluidic channel design.

4. Video clip 2_ Zoomed in Printing

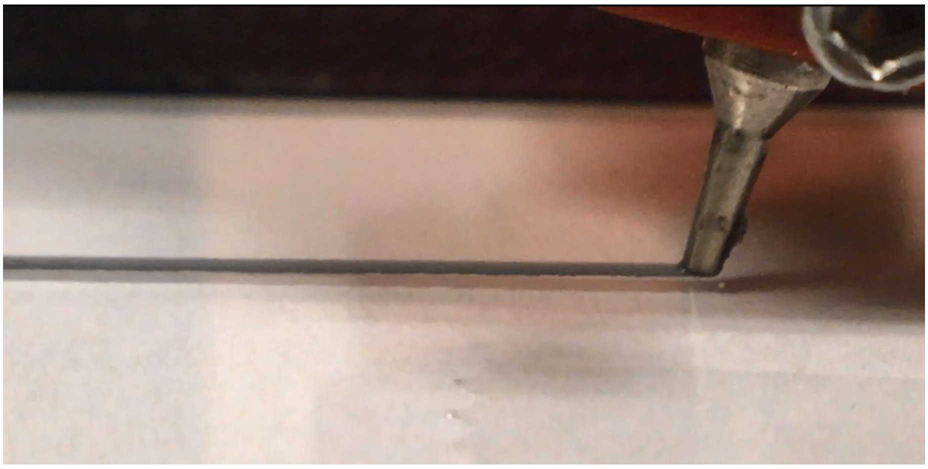

Figure S4 A screen capture from the video clip 2 (Video S2), in which a zoomed in view of the contact based printing method, laying down wax on a glass substrate. Note both the extruder tip geometry and angle relative to the substrate.

5. Cost analysis of wax based technique vs. soft lithography

A preliminary cost analysis was done to compare the two techniques, first from an equipment start-up cost, and second by a unit device cost. As shown in Table I, the proposed wax based fabrication technique is significantly less expensive to not only set up, but also operate. It eliminates the need for expensive hazardous chemical such as hydrofluoric acid, photoresist, and developer. These are all chemicals that need to be replenished over time. From a unit device cost, as shown in Table II, the costs are comparable. While traditional soft lithography has resolution and better uniformity over the wax based technique, the initial equipment costs, continued costs of perishables, and operational complexity creates a high barrier to entry for researchers to prototype designs and devices. At a sub $1,000.00 price point, the wax based technique opens up microfluidic device fabrication to a larger number of researchers, students, and/or developing population groups.

**Table S1: Start-up Costs Associated with Microfluidic Device Fabrication**			
**Wax Based Technique**		**Traditional Soft Lithography**	
Wax Printer	$450.00	Photolithography Machine	$60,000
Cooling Bed	$400.00	Photoresist	$800.00
Heating Element	$80.00	Developer	$100.00
Extruder Tips	$23.00	Hotplate	$1000.00
Extension cables	$7.00	Spin Coater	$6,000.00
		Masks	$125.00
**Total**	**$960.00**		**$68,025.00**
Costs based upon current University Equipment. Price subject to vary			

**Table S2: Low Volume Device Unit Costs (in USD)**			
**Wax Based Technique**		**Traditional Soft Lithography**	
Glass Slide	$0.20	Glass Slide	$0.20
PDMS	$1.00	PDMS	$1.00
Wax	$0.03		
**Total**	**$1.23**		**$1.20**
